# Foot force direction control during a pedaling task in individuals post-stroke

**DOI:** 10.1186/1743-0003-11-63

**Published:** 2014-04-16

**Authors:** Jing Nong Liang, David A Brown

**Affiliations:** 1 Department of Physical Therapy and Human Movement Sciences, Suite 1100, 645 N. Michigan Avenue, Chicago, IL 60611, USA; 2 Interdepartmental Neuroscience Program, Feinberg School of Medicine, Northwestern University, 645 N. Michigan Ave. Suite 1100, Chicago, IL 60611, USA; 3 Department of Physical Therapy, School of Health Related Professions, University of Alabama at Birmingham, 1720 2nd Ave S, Birmingham, AL 35233, USA

**Keywords:** Stroke, Locomotion, Force control, Muscle activity, Pedaling

## Abstract

**Background:**

Appropriate magnitude and directional control of foot-forces is required for successful execution of locomotor tasks. Earlier evidence suggested, following stroke, there is a potential impairment in foot-force control capabilities both during stationary force generation and locomotion. The purpose of this study was to investigate the foot-pedal surface interaction force components, in non-neurologically-impaired and stroke-impaired individuals, in order to determine how fore/aft shear-directed foot/pedal forces are controlled.

**Methods:**

Sixteen individuals with chronic post-stroke hemiplegia and 10 age-similar non-neurologically-impaired controls performed a foot placement maintenance task under a stationary and a pedaling condition, achieving a target normal pedal force. Electromyography and force profiles were recorded. We expected generation of unduly large magnitude shear pedal forces and reduced participation of multiple muscles that can contribute forces in appropriate directions in individuals post-stroke.

**Results:**

We found lower force output, inconsistent modulation of muscle activity and reduced ability to change foot force direction in the paretic limbs, but we did not observe unduly large magnitude shear pedal surface forces by the paretic limbs as we hypothesized.

**Conclusion:**

These findings suggested the preservation of foot-force control capabilities post-stroke under minimal upright postural control requirements. Further research must be conducted to determine whether inappropriate shear force generation will be revealed under non-seated, postural demanding conditions, where subjects have to actively control for upright body suspension.

## Background

Successful execution of a locomotor task requires appropriate magnitude and directional control of foot-forces (i.e. exerted by the foot on a stable surface). For example, during upright walking, leg muscles must exhibit appropriate coordination that results in adequate foot-force amplitude and direction at the end of the stance phase to propel the center of mass forward, at initial contact to brake the forward acceleration of the center of mass, and during mid-stance to support the center of mass and prevent collapse of the stance limb [1].

Previous human locomotion studies suggested that individuals with post-stroke hemiplegia exhibit impaired foot force control capabilities [2,3]. For example, during steady state upright walking, the anterior-posterior ground reaction force patterns are typically bilateral and symmetric with a reversal from breaking at initial contact to propulsion at mid-stance of gait. However, for individuals with hemiparesis, asymmetries in forces between the limbs have been observed, with horizontal forces that were generally characterized by higher breaking forces during initial contact and reduced propulsive forces at terminal stance [3]. Using a pedaling paradigm, Rogers et al. [2] reported impaired foot-force control capabilities in post-stroke individuals, with respect to both force magnitude and direction. When pushing on a stationary pedal and during dynamic pedaling, a shift in force path orientation was observed in the paretic limbs, rotating it anteriorly away from the hip, suggesting that the paretic leg had a different preferred direction of force generation compared to the non-paretic leg. These observations imply a potential impairment in appropriate vector force direction control during locomotion in individuals post-stroke. However, these studies did not account for the impaired ability of the paretic leg in force output [4,5], making it difficult to derive an appropriate comparison of force vector components.

Due to the advantages afforded by the pedaling paradigm to minimize postural upright control requirements, to specify a consistent kinematic and kinetic trajectory for the task, and the ability to achieve foot force control tasks even with neurologically-impaired individuals, we extended the work of Rogers et al., to examine the control of foot-forces during a pedaling task that required some explicit directional foot-force control. For a pushing task on a pedal or a dynamic pedaling task, the control of foot placement on a pedal surface is maintained when normal pedal forces are generated, yet is destabilized (i.e. tend to slip forward or backward) when fore-aft shear forces are generated. The control problem associated with maintaining normal surface forces while minimizing fore-aft shear forces is common, for example when the surface is of low friction, inappropriate generation of excessive shear or horizontal forces could result in a slip. The post-stroke nervous system may not have the inherent flexibility to activate muscles in a selective manner that allows control of normal surface forces when shear surface forces needs to be constrained, such as when environmental demands are encountered. The purpose of this current study was to investigate the foot-pedal surface interaction force components in non-impaired and stroke-impaired individuals in order to determine how fore/aft shear-directed foot/pedal forces are controlled when subjects attempt to generate target normal pedal surface forces. We were interested in this problem, because we hypothesized that the stroke-impaired nervous system, due to its reduced selectivity in activating muscles [6-9], is impaired in the ability to regulate fore-aft shear forces when trying to control a foot placement maintenance task, such as generating a target normal pedal force magnitude during a static push task and during a dynamic pedaling task. We expected that the control of a stabilizing task would result in the generation of unduly large force magnitudes in the shear pedal surface direction and in reduced participation of multiple muscles that can contribute forces in the appropriate direction.

## Methods

### Subjects

Sixteen individuals with chronic post-stroke hemiplegia [age (mean ± SD) = 62.9 ± 8.7 years; 183.1 ± 87.7 months post-stroke] and 10 age-similar non-neurologically impaired individuals [age = 62.2 ± 11.0 years] participated in this study. Inclusion criteria for participants post-stroke were single, unilateral stroke ≥12 months before the study, lower-limb hemiparesis with no lower extremity contractures, no major cognitive, perceptual, sensory deficits or acute cardiovascular impairments. Ambulatory ability ranged from independent ambulation without assistive devices to independent ambulation with cane/quad-cane. For subjects with an ankle-foot orthosis, it was removed during data collection. Each participant received written and verbal information about the experiment procedures before giving written consent. The protocol was approved by the Institutional Review Board at Northwestern University.

### Instrumentation

**Figure 1:**
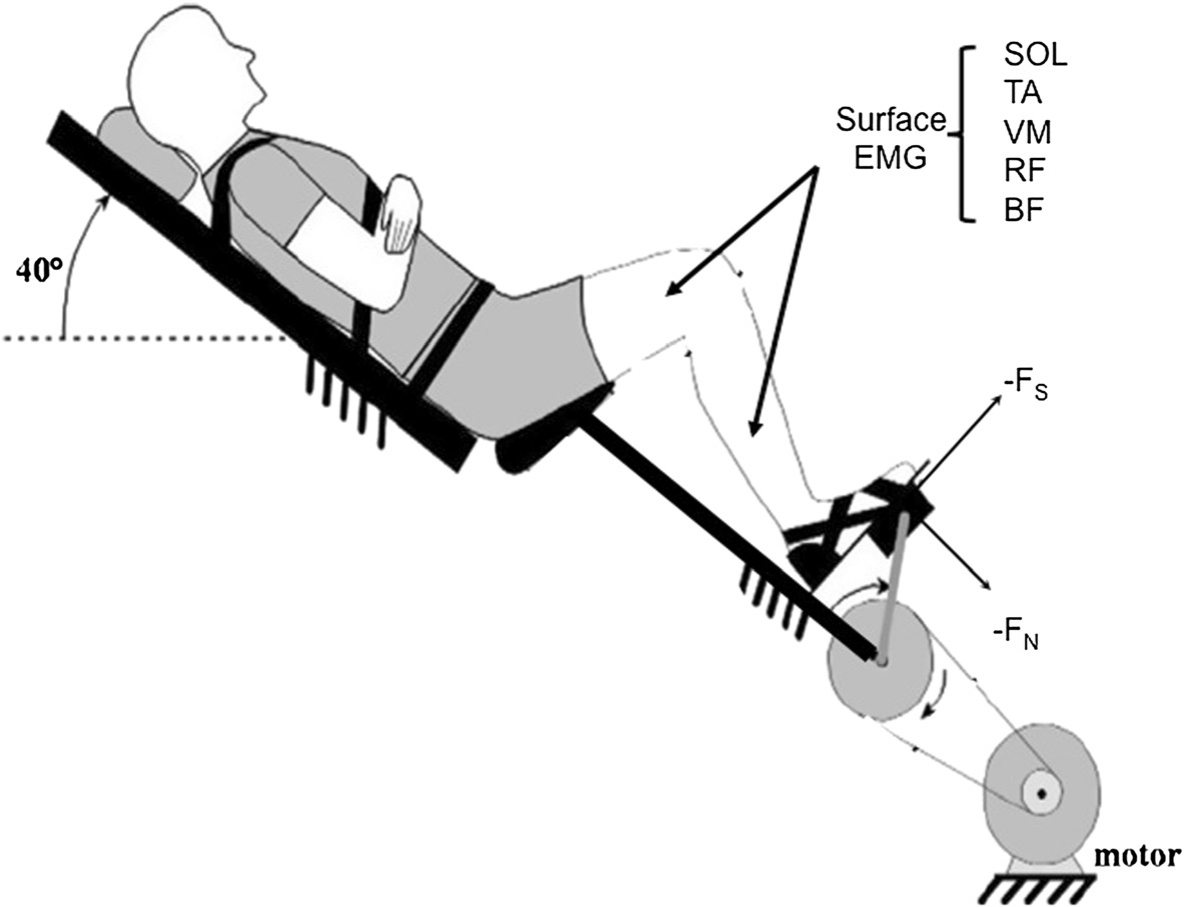
**Participants were seated on the seat with trunk stabilized onto the backboard which was kept tilted 40° from horizontal.** The motor held the crank at 90° from top dead center during the fixed-crank conditions, and moved the crank at a constant velocity of 40 rpm during moving-crank conditions. All tasks were performed with the test leg only, while the other leg rested on stable surface throughout the experiment.

**Figure 2:**
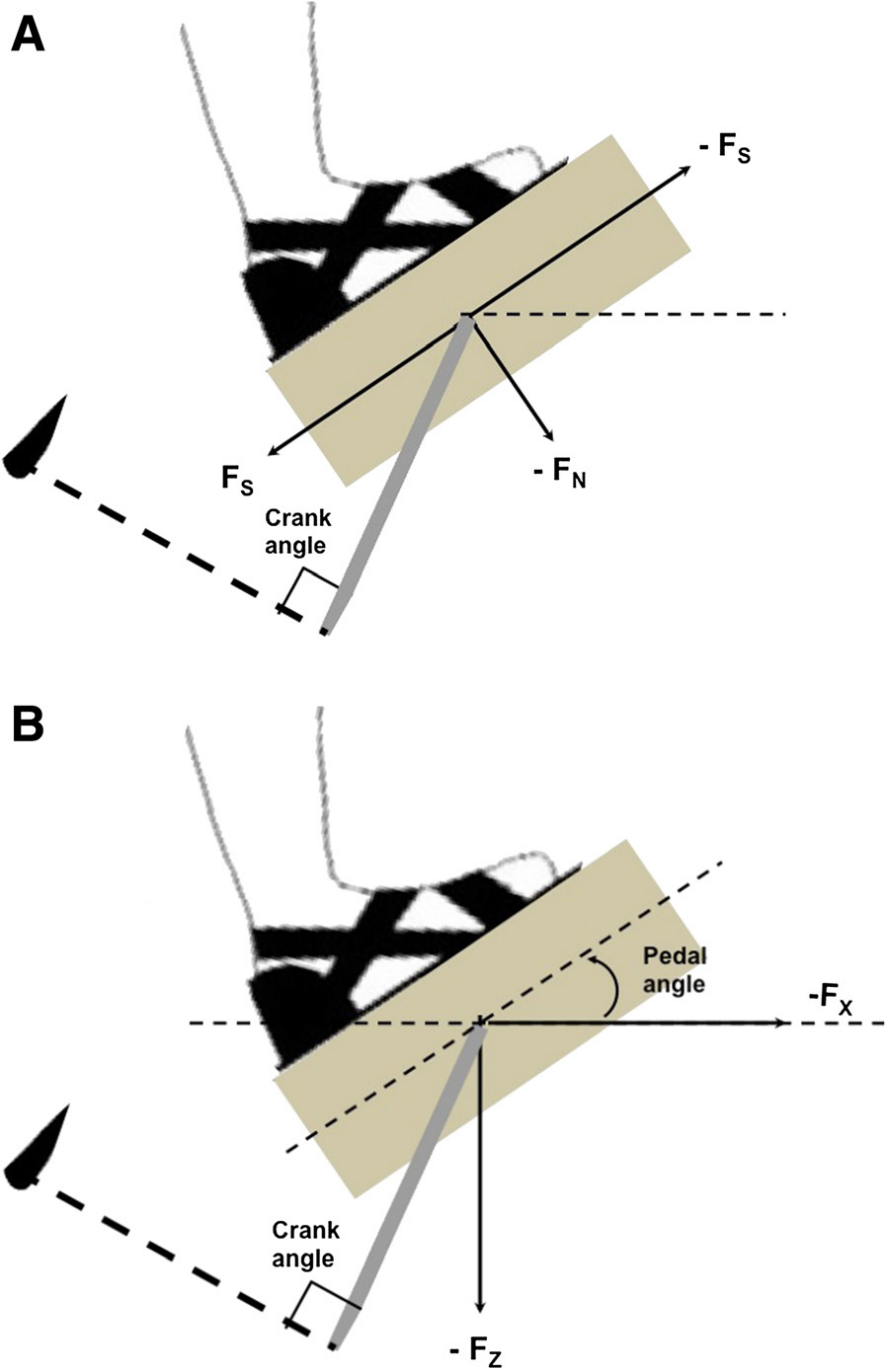
**Two coordinate systems were defined: the pedal coordinates system and the global coordinates system. (A)** The pedal reference system was originally set to allow the F_N_ (normal force perpendicular to pedal) and F_S_ (shear force parallel to pedal) exerted on the pedal to be defined, with negative normal force directed downwards (F_N_), and negative shear force (F_S_) directed towards the front of the pedal. Then, a 2-D rotation matrix was used to transfer the data initially obtained with respect to the pedal reference system to the global coordinate system **(B)**, to allow the forces perpendicular (F_Z_) and parallel (F_X_) to the horizontal ground and the Ped_Ang_ orientation relative to the horizontal ground to be computed, with negative vertical forces (F_Z_) directed downwards and negative horizontal forces (F_X_) directed towards the front of the cycle ergometer. Pedal angle position (Ped_Ang_) was expressed with respect to horizontal.

A custom-made cycle ergometer (Figure 1) with instrumented pedals, a seat with backrest and a motor-driven crank was used for this study [2]. Participants were seated on the seat with the torso stabilized against the backrest (inclined 40° from horizontal) to maintain constant hip position. All movements were confined to the legs throughout the experiment. Optical encoders (BEI model EX116-1024-2), one at each pedal spindle and one coupled to the right crank, provided measurements of the pedal angles and the crank position with ±0.3° accuracy. Force transducers in each pedal measured the three-dimensional foot/pedal force vector (Delta 660, ATI-IA Inc, Garner, NC) (Figure 2). Pedaling velocity was controlled by an electric motor (12:1 gear reducer, 3.7 hp, model MT506B1-S1C1, Kollmorgen, Radford, VA) and was kept constant at 40 revolutions/minute (rpm) for all subjects during the moving-crank task.

Bipolar silver surface electrodes (DelSys, 1 cm length, 1 mm wide, 1 cm inter-electrode distance) were used to record the electromyograms (EMG) from five muscles on the test leg: vastus medialis (VM), rectus femoris (RF), biceps femoris (BF), soleus (SOL) and tibialis anterior (TA). EMG signals were amplified with a gain of 10 at the electrode site before remote differential amplification (CMRR: 92 dB, gain range 100–1000 times, frequency response 20-450 Hz) and were low pass filtered (custom-designed filter, 500 Hz cutoff). The signals from the optical encoders and force-transducers were converted from digital to analog with a D/A converter module before sampling. All signals were then sampled at 1000 Hz via a 12-bit A/D converter (National Instruments) and custom LabView software.

### Experimental protocol

All trials were performed by one leg (test leg), while the opposite leg rested in a static 90/90, hip/knee position on a stable surface. While seated on the bicycle seat, participants pushed against the pedal fixed in the middle of the downstroke to generate 3 maximal force efforts. Real-time visual feedback of the target F_N_ to be achieved, which was 40 ± 5% of the individual’s mean maximal effort F_N_, was provided on a monitor in the form of a bar graph and subjects were instructed to push against the pedal until the bar representing the F_N_ generated met the target line for a success trial. Each participant performed this foot placement maintenance task achieving the target F_N_ under 2 conditions: fixed-crank at 90°, and crank moving at 40 rpm.

#### Fixed-crank task

Participants were first instructed to achieve target F_N_ with no regard for foot orientation, i.e. “regular” push. Then, in order to obtain a wide range of pedal shear force (F_S_) values, subjects assumed a variety of foot orientations (from most dorsiflexed to most plantarflexed) on the pedal. With the crank fixed at 90°, participants moved their foot position to meet a target pedal angle position (Ped_Ang_) displayed on an oscilloscope, while simultaneously achieving their respective target F_N_. Each subject attempted a number of target Ped_Ang_ , determined by dividing the range of maximum voluntary ankle dorsiflexion and plantarflexion postures into divisions of 7°, yielding several target Ped_Ang_ per subject. Each subject generated a minimum of 2 to a maximum of 12 Ped_Ang_ depending on the person’s ankle active range. In the most plantarflexed position, F_S_ were anteriorly-directed, whereas in the most dorsiflexed position, F_S_ were less anteriorly-directed or even posteriorly-directed. The Ped_Ang_ were presented to the subject in a randomized order and repeated 3 times.

#### Moving-crank task

With the motor-driven crank moving at 40 rpm, participants were instructed to pedal along for 30 seconds, keeping the F_N_ output within the range displayed. The task was repeated for 3 different conditions: (1) regular pedaling – with no regard for Ped_Ang_, (2) pedaling with sustained maximal active ankle dorsiflexion (less anteriorly-directed F_S_), and (3) pedaling with sustained maximal active ankle plantarflextion (higher anteriorly-directed F_S_). Each condition was repeated 3 times.

### Data analysis

All force and EMG data were processed using custom MATLAB programs. Two coordinate systems were defined: the pedal (Figure 2A) and the global coordinate systems (Figure 2B), to analyze pedal forces. The pedal reference system was originally set to allow the F_N_ (normal force perpendicular to pedal) and F_S_ (shear force parallel to pedal) exerted on the pedal to be defined. Then, a 2-D rotation matrix was used to transfer the data initially obtained with respect to the pedal reference system to the global coordinate system, to allow the forces perpendicular (F_Z_) and parallel (F_X_) to the horizontal ground and the Ped_Ang_ orientation relative to the horizontal ground to be computed.

#### Fixed-crank

For each Ped_Ang_, the magnitude of F_S_ and F_N_ were averaged over a 1-second period when a steady, consistent amount of target F_N_ was generated. To normalize the F_S_ magnitude between subjects with different force generation capabilities, we calculated the ratio of F_S_ per unit F_N_ (F_S_/F_N_ ratio). Similarly, to normalize the F_X_ magnitude between subjects with different force generation capabilities we calculated the ratio of F_X_ per unit F_Z_ (F_X_/F_Z_ ratio). The EMG for each muscle were rectified and integrated over the corresponding 1-second period.

During regular push, when subjects pushed against the pedal achieving target F_N_, each variable in both pedal (F_N_, F_S_/F_N_ ratio) and global coordinates (F_Z_, F_X_/F_Z_ ratio, Ped_Ang_) were averaged over the 3 attempts performed. We used an independent t-test, to compare between the 2 groups (non-impaired vs post-stroke impaired). When the subjects were tested with a wide variety of Ped_Ang_, we performed a linear regression analysis for each individual to examine the contributions of Ped_Ang_ in variability of F_S_/F_N_ and F_X_/F_Z_ , respectively. Slopes of the regression lines were compared between groups using an independent t-test.

To determine the contribution of Ped_Ang_ and EMG activity on F_X_/F_Z_, a stepwise regression was performed. Another multiple linear regression analysis using only the resulting Ped_Ang_, VM, RF model was then conducted.

#### Moving-crank

We averaged the F_N_ profiles over the 20 cycles sampled. The crank-angle position at which the peak target F_N_ was achieved was used as an index to identify the corresponding F_S_ value. EMG signals were integrated for the entire downstroke phase (0º ~ 180° of the crank cycle with respect to top-dead-center) and averaged across cycles. All EMG profiles were smoothed with a fourth-order, zero-lag, low-pass Butterworth filter with a cutoff frequency of 25 Hz. EMG activity for each muscle that occurred when subjects performed sustained ankle dorsiflexion and plantarflexion pedaling was expressed as a percent change from dorsiflexed to plantarflexed conditions. As with fixed-crank conditions, during regular pedaling, sustained maximal dorsiflexion and plantarflexion pedaling, the variables in both pedal (F_N_, F_S_/F_N_ ratio) and global coordinates (F_Z_, F_X_/F_Z_ ratio, Ped_Ang_) were averaged over the 3 attempts. Using independent t-tests, we compared each variable between the 2 groups for each condition. For EMG statistical analysis, one-way ANOVA was conducted for each muscle, comparing the percent change in EMG amplitude from the dorsiflexed to the plantarflexed condition. For all analyses, alpha-level was set at 0.05.

## Results

### Fixed-crank

During the regular push task, when achieving the targeted F_N_, individuals post-stroke generated smaller magnitudes of target F_N_ (21.9% less) compared with non-impaired individuals (p < 0.05). Despite this lower target F_N_ magnitude, we did not observe a difference in F_S_/ F_N_ between the 2 groups (p > 0.05). However, relative to global coordinates, individuals post-stroke generated less anteriorly-directed F_X_/F_Z_ (p < 0.05) (Table 1). We also did not observe a difference in Ped_Ang_, between post-stroke (30.8° upward rotation from horizontal) and non-impaired (33.4° upward rotation from horizontal) individuals (p > 0.05).

**Table 1:** **Comparison of forces (mean ± SE) in pedal (F**_**S**_**/F**_**N**_**) and global (F**_**X**_**/F**_**Z**_**) coordinate systems during the regular push (fixed-crank) and regular pedaling (moving-crank) tasks**

**Task**	**Group**	**Target F**_**N **_**(N)**	**F**_**S**_**/F**_**N**_	**F**_**X**_**/F**_**z**_
Fixed	Non-Impaired	209.4±11.3	0.26±0.07	1.13±0.07
Crank	Post-Stroke	163.5±8.9*	0.13±0.05	0.79±0.05*
Moving	Non-Impaired	220.6±13.1	0.19±0.04	1.60±0.14
Crank	Post-Stroke	161.1±8.5*	0.16±0.08	1.27±0.10

*Denotes statistical significance when compared with non-impaired group with p < 0.05.

Target F_N_ is 40% of maximal effort F_N._

When subjects were asked to match target F_N_ while simultaneously assuming a large range of different target Ped_Ang_, the Ped_Ang_ accounted for much of the variance in the F_S_/F_N_ that was generated for both post-stroke (R^2^ = 0.87, slope = −0.015, p < 0.05) and non-impaired individuals (R^2^ = 0.96, slope = −0.025, p < 0.05), with no observed difference in the regression slopes between the 2 groups (p > 0.05). To determine if this relationship between F_S_/ F_N_ and Ped_Ang_ was indicative of a more global strategy to maintain a preferred direction of force, we examined this relationship in the global coordinates system. Relative to global coordinates, Ped_Ang_ was still able to account for much of the variance in F_X_/F_Z_ generated in non-impaired individuals (R^2^ = 0.77, slope = 0.01, p < 0.05), indicating a change in global force direction with different Ped_Ang_, but not in individuals post-stroke (R^2^ = 0.37, slope = 0.001, p > 0.05), showing consistent global force direction regardless of Ped_Ang_.

With individuals post-stroke, when using Ped_Ang_, SOL, TA, VM, RF and BF muscle activity as independent variables in a stepwise regression model to examine the relationship of EMG activity with horizontally-directed F_X_, we did not observe any consistent contribution of a single variable, whereas in non-impaired individuals, Ped_Ang_ was a consistent inclusion in all the subjects, and VM and RF muscles were included in >50% of the subjects. Further regression analysis using the Ped_Ang_, VM and RF model indicated that together, they accounted for, on average, 90% of the variance in F_X_ in non-impaired individuals. However, the same model could only explain 76% of the variance in 11/16 post-stroke individuals, with 5 subject’s variability in F_X_ not explainable by this model.

### Moving crank

During regular pedaling, individuals post-stroke generated smaller magnitude of target F_N_ (27% less) compared with non-impaired individuals (p < 0.05), and we did not observe a difference in F_S_/F_N_ between the 2 groups (p > 0.05), similar to fixed-crank conditions (Table 1). Although we did not observe a statistically significant difference in F_X_/F_Z_ when comparing post-stroke to non-impaired data, the values trended toward significance (p = 0.08), but we were severely underpowered for this particular comparison due to the large variance in this variable. As with the fixed-crank task, we did not observe any difference in Ped_Ang_ between post-stroke (42.5° upward rotation from horizontal) and non-impaired (45.8° upward rotation from horizontal) individuals during regular pedaling (p > 0.05).

When subjects pedaled with sustained maximal ankle dorsiflexion, achieving target F_N_, individuals post-stroke generated smaller magnitudes of target F_N_ (26.9% less) (p < 0.05) and we did not observe a difference in F_S_/F_N_ between the 2 groups (p > 0.05), similar to regular push and regular pedaling conditions. However, relative to global coordinates, individuals post-stroke generated less anteriorly-directed F_X_/F_Z_ when pedaling with sustained maximal ankle dorsiflexion (p < 0.05), accompanied by a smaller upward rotation angle (43.5° upward rotation from horizontal) compared to non-impaired individuals (60.1° upward rotation from horizontal) (p < 0.05).

When subjects pedaled with sustained maximal ankle plantarflexion, achieving the target F_N_, individuals post-stroke generated smaller magnitudes of target F_N_ (26.6% less) compared with non-impaired individuals (p < 0.05). We did not observe a difference in F_S_/F_N_, F_X_/F_Z_ and Ped_Ang_ between the 2 groups (p > 0.05).

**Figure 3:**
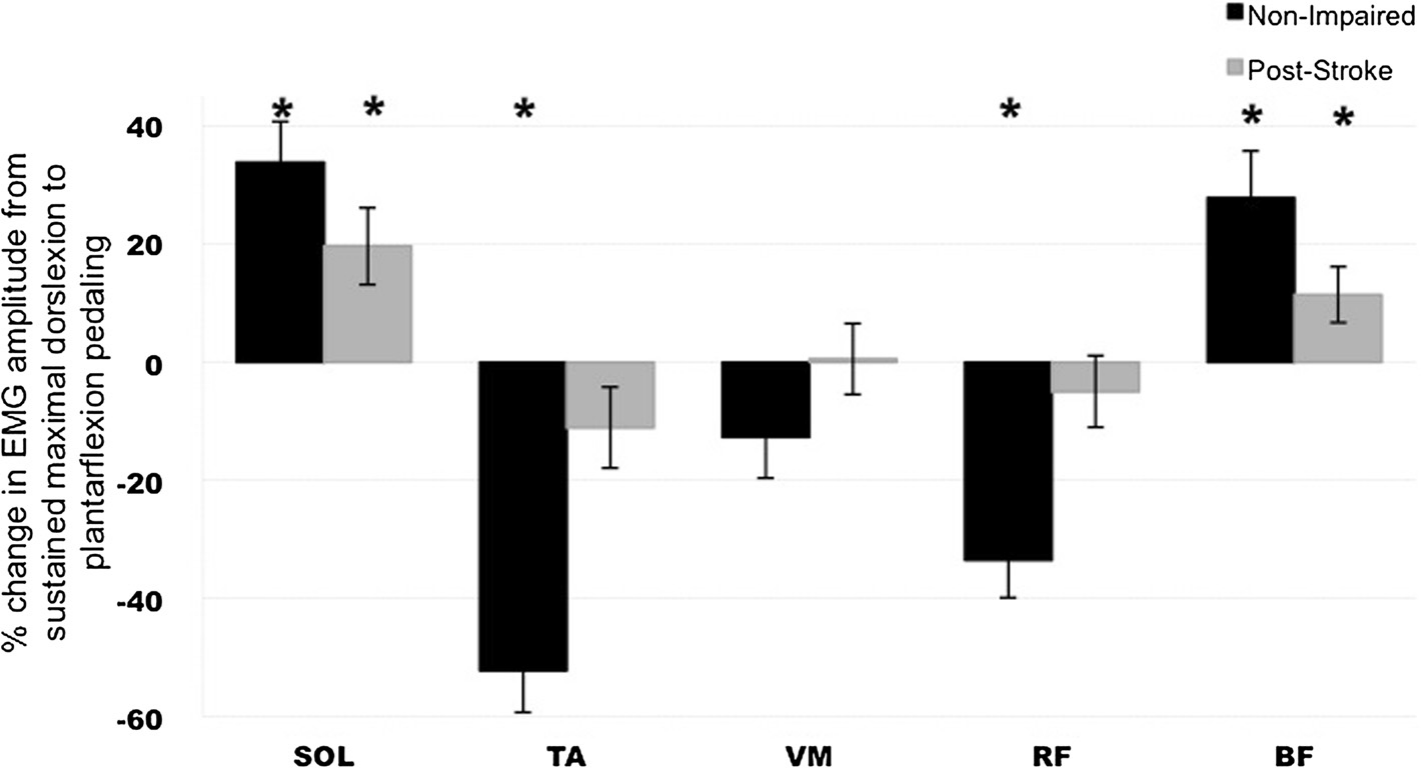
**Percentage change in EMG amplitude (mean ± SE) when the subject pedals with sustained maximal ankle dorsiflexion to plantarflexion for the SOL, TA, VM, RF and BF muscles.** *denotes statistically significant change between sustained maximal ankle dorsiflexion to plantarflexion conditions (p < 0.05). A positive value indicates an increase in the EMG amplitude and a negative change indicates a decrease.

In non-impaired subjects, when comparing the percent change in muscle activity during pedaling with sustained maximal ankle plantarflexion relative to dorsiflexion, we observed increased SOL (p < 0.05), decreased TA (p < 0.05), decreased RF (p < 0.05) and increased BF (p < 0.05) muscle activity amplitudes, with no change in VM activity. In individuals post-stroke, we observed increased SOL (p < 0.05) and increased BF (p < 0.05) amplitudes only, with no changes in TA, VM and RF muscles (Figure 3).

## Discussion

We hypothesized that the stroke-impaired nervous system would be impaired in its ability to regulate fore-aft shear forces when trying to control a foot stability maintenance task. Our hypotheses were only partially supported. Although we did not observe unduly large shear pedal surface forces in subjects post-stroke, we did observe lower force output, inconsistent modulation of paretic muscle activity and reduced ability to change foot-force direction by adjusting foot posture resulting in a relatively fixed direction of foot-forces generated relative to global coordinates, when compared to non-impaired individuals, who demonstrated paralleled changes in foot-force directions when foot position was altered.

We observed smaller force output by the paretic leg, quantified as the target F_N_ which was 40% of the maximal effort F_N_, compared to non-impaired individuals, during both the fixed and moving-crank tasks. This was consistent with previous literature that documented loss of capacity to generate high muscle force levels following stroke, likely as a result of muscle disuse atrophy and reduced descending commands post-stroke [4,5]. It has been suggested that impairments in voluntary force production post-stroke is more likely due to altered descending commands, rather than muscle atrophy [10].

We observed a lack of active TA muscle involvement when individuals post-stroke pedaled with sustained maximal dorsiflexion and plantarflexion, compared to controls. During locomotor tasks, reduced active ankle dorsiflexion of the paretic leg during swing has been commonly observed during gait [11]. Despite the lack of TA muscle activity, the paretic ankle Ped_Ang_ positions were comparable to the non-impaired ankles in the attempt to achieve the target F_N_. In the attempt to achieve target F_N_ both during fixed-crank and the moving-crank locomotor tasks, the ankle was likely passively moved into dorsiflexion during force-exertion against the slowly moving crank, suggesting that this positioning did not require ankle muscle activity. The paretic ankle joint angular displacement has also been reported to be similar to non-paretic ankles during seated pedaling [12].

During fixed-crank conditions, non-impaired individuals varied the horizontal forces generated by adjusting the Ped_Ang_, VM and RF variables. This model did not consistently occur in the post-stroke group. Similarly, during moving-crank conditions, we observed the lack of paretic TA and RF muscle involvement. Earlier studies reported in healthy controls, during both standing [13] and walking tasks [14], combinations of different single muscle activities were variably recruited in flexible combinations of muscle modules relative to the task, indicating a general neural strategy in the non-impaired nervous system. However, in stroke-impaired nervous system, fewer muscle modules were recruited, and a less complex coordination pattern was observed during walking [15], suggesting that a disruption in descending pathways resulted in decreased muscular independence and flexibility.

Surprisingly, we did not observe unduly high shear or horizontal forces by paretic limbs. In both fixed and moving-crank conditions, normalized shear and horizontal force values were comparable, if not lower, than non-impaired limbs. In retrospect, this may be attributed to the nature of our task, involving minimal postural control, as the upper body was fully supported. Thus, there was less need to control upright posture, therefore, the nervous system was better able to adopt compensatory strategies that can allow the ankle to move into the preferable postures in the attempt to achieve target F_N_. Evidence suggested that addition of postural control could interfere with locomotor output in people post-stroke during a standing, non-seated pedaling task [16]. Thus, further research must be conducted to determine whether inappropriate shear forces will be revealed under non-seated, postural demanding conditions, where subjects have to actively control for posture [17].

### Limitations

We recognize that the pedaling model used to study locomotion is not the same as walking, and generalizing our results to walking conditions is limited. Nevertheless, the line of research that has been conducted in our lab and some earlier research reporting similarities in post-stroke muscle activity patterns during pedaling and walking [18,19] suggests pedaling to be an appropriate model for studying basic neural control mechanisms of locomotion under reproducible biomechanical conditions that allow comparisons between impaired and non-impaired subjects. In addition, we also recognize that the task performed in the present study was a one-legged task. If there had been non-paretic limb involvement, we could have examined the compensation of the non-paretic limb for the paretic limb control problem, if any. While this design does not take into account any potential non-paretic limb influences, it nevertheless provides a more controlled approach, which allowed us to interpret the force control capabilities of the paretic limb.

Another limitation was that some of our post-stroke participants had limited active ankle range of motion, and thus could only generate limited levels of pedal angle positions. To accommodate for inter-individual variability, we recruited more participants in the post-stroke group to better capture the range of characteristics post-stroke.

### Clinical implications

The impaired ability of the post-stroke nervous system to actively control ankle position contributed to the impaired ability to regulate horizontal surface forces. Inadequate amounts of horizontal surface forces could produce negative consequences during gait; insufficient amounts could lead to inefficient propulsion, or excessive amounts could result in the foot slipping off the support surface.

## Competing interests

The authors declare that they have no competing interests.

## Authors’ contributions

JL carried out the recruitment of participants, data collection, data analysis, statistical analysis and drafted the manuscript. DB participated in statistical analysis and drafted the manuscript. All authors read and approved the final manuscript.

## Authors’ information

JL was supported by the American Heart Association Predoctoral Award #11PRE5430029.
